# Psychiatric safety of methylphenidate in adults with major depressive disorder: a 1-year retrospective cohort study of 6,422 patients

**DOI:** 10.1017/S0033291726103845

**Published:** 2026-05-05

**Authors:** Ting-Hui Liu, Ya-Lin Huang, Jheng-Yan Wu, Chien-Ho Lin, Fong-Lin Jang, Chih-Cheng Lai

**Affiliations:** Chi Mei Medical Center, Taiwan

**Keywords:** depressive disorder, drug-related side effects and adverse reactions, hospitalization, major, methylphenidate, suicidal ideation

## Abstract

**Background:**

Methylphenidate is sometimes used to address residual symptoms of major depressive disorder (MDD), but concerns about psychiatric destabilization and limited long-term evidence have constrained its use. We examined the psychiatric safety of methylphenidate in adults with MDD in a large, real-world cohort.

**Methods:**

Using the TriNetX Global Collaborative Network, we identified adults with MDD who initiated methylphenidate and matched them 1:1 with controls who did not receive methylphenidate. Patients with attention-deficit/hyperactivity disorder, bipolar disorder, mania, or recent psychiatric destabilization were excluded. The primary outcome was a composite of all-cause hospitalization or emergency room visits; secondary outcomes included hospitalization, emergency visits, suicidal behavior, manic episodes, and recurrence of MDD. Hazard ratios (HRs) were estimated with Cox proportional hazards models after propensity score matching.

**Results:**

Of 425,190 eligible patients, 3,211 matched pairs were included (mean age, 55.8 years; 58% female). Over 1 year, the composite outcome occurred less frequently in the methylphenidate group than in controls (574 vs. 694; HR, 0.85; 95% CI, 0.76–0.95). No significant differences were observed for hospitalization, emergency visits, suicidal behavior, manic episodes, or MDD recurrence. Results were consistent across subgroups defined by sex, age, and antidepressant class.

**Conclusions:**

In adults with MDD, methylphenidate use was associated with a lower risk of hospitalization or emergency visits and was not linked to increased risk of suicidality, mania, or recurrence. These findings support the psychiatric safety of methylphenidate as an adjunctive treatment for selected patients, though longer follow-up is needed.

## Introduction

Major depressive disorder (MDD) remains a leading cause of disability worldwide, with a substantial proportion of patients failing to achieve full remission despite first-line antidepressant therapy. Indeed, the STAR*D trial and subsequent large-scale studies have demonstrated that up to half of patients do not respond adequately to their first antidepressant, and many continue to experience residual symptoms such as fatigue, apathy, impaired concentration, and psychomotor slowing (Al-Harbi, 2012; Rush et al., 2004). These residual symptoms are clinically meaningful because they are associated with poor functional outcomes, diminished quality of life, and a higher risk of relapse or hospitalization (Nierenberg, 2015).

In clinical practice, psychostimulants such as methylphenidate have occasionally been prescribed as adjunctive treatments in patients with treatment-resistant depression or those with disabling residual symptoms (Bahji & Mesbah-Oskui, 2021; Kerr et al., 2012). Early clinical reports and open-label trials suggested that stimulant augmentation may offer rapid improvements in mood, energy, and cognition (Lavretsky et al., 2015a, 2015b). However, concerns regarding psychiatric destabilization, particularly treatment-emergent mania, increased suicidality, and potential for misuse, have limited the widespread adoption of this strategy (Chakraborty & Grover, 2011; Oliva et al., 2025). Most of the available evidence comes from small-sample studies or short-term randomized controlled trials, with follow-up durations typically limited to 4–12 weeks (Pary et al., 2015; Patkar et al., 2006; Ravindran et al., 2008). Thus, questions remain about whether methylphenidate is safe to use in patients with MDD over longer periods and in real-world populations.

To address these gaps, we conducted a large-scale cohort study using the TriNetX Global Collaborative Network to evaluate the psychiatric safety of methylphenidate use in patients with MDD. Our aim was to determine whether methylphenidate is associated with increased risks of hospitalization, emergency visits, suicidality, mania, or illness recurrence, and to provide real-world evidence on its potential role as an adjunctive treatment for residual depressive symptoms.

## Methods

### Data sources

This retrospective cohort study was conducted using the TriNetX Global Collaborative Network, a global federated health research network of de-identified electronic health records (EHRs) TriNetX. This network aggregates data from a diverse range of healthcare organizations (HCOs), including academic medical centers, nonacademic hospitals, and specialist centers. Available data include demographics, diagnoses, medications, procedures, and encounters, and are provided in aggregate form through built-in analytic tools. Individual-level data were not accessible due to platform policies. Further details on data access are available at https://trinetx.com (‘TriNetX’). Written informed consent was not required, as all data were anonymized.

### Patient selection

Adult patients (aged ≥18 years) with a diagnosis of MDD were identified based on ICD-10 codes. Patients with a diagnosis of attention-deficit/hyperactivity disorder (ADHD) were excluded from both cohorts to eliminate confounding by indication.

### Methylphenidate group

The methylphenidate group included adults with MDD who received a prescription for methylphenidate on or before June 1, 2024. An MDD diagnosis was required within 1 month before the first methylphenidate prescription, along with at least one antidepressant prescription in the same period to reflect augmentation therapy. Patients were excluded if they had any prior diagnosis of ADHD, bipolar disorder, or mania, or if they had used mood stabilizers such as lithium, valproate, or carbamazepine. Those with psychiatric destabilization, defined as inpatient admission, suicidal ideation, self-harm, or suicide attempt occurring between 3 and 1 months before the index date, were also excluded. To ensure incident use, patients with prior methylphenidate exposure were removed. All patients were required to have had an inpatient encounter within 1 month before the index date to establish comparable baseline severity. The complete definitions of criteria are presented in Supplementary Table 1.

### Control group

The control group included adults with MDD who received an antidepressant prescription on or before June 1, 2024, without any history of methylphenidate use. Exclusion criteria were the same as for the control group. These included any prior diagnosis of ADHD, bipolar disorder, or mania, as well as prior use of mood stabilizers, including lithium, valproate, or carbamazepine. Evidence of psychiatric destabilization, defined as inpatient admission, suicidal ideation, intentional self-harm, or suicide attempt occurring between 3 months and 1 month before the index date, was also excluded. All patients were required to have had an inpatient encounter within 1 month before the index date to ensure comparable baseline severity. Full diagnostic code definitions for cohort selection are detailed in Supplementary Table 1.

### Index date

The index date for the methylphenidate group was defined as the first prescription of methylphenidate on or before June 1, 2024, with an MDD diagnosis recorded within 1 month prior. For the control group, the index date was the date of antidepressant prescription on or before June 1, 2024, also requiring an MDD diagnosis within the prior month. Full diagnostic criteria for MDD and index event definitions are provided in Supplementary Table 2.

### Covariates

Covariates included age, sex, race, medical comorbidities, psychiatric diagnoses, psychotropic medications, and healthcare utilization before the index date.

Medical conditions included hypertension, type 2 diabetes, hyperlipidemia, ischemic heart disease, chronic kidney disease, and chronic obstructive pulmonary disease. Psychiatric conditions included anxiety disorders, post-traumatic stress disorder, intellectual disability, and substance use disorders, including alcohol, opioids, cannabis, stimulants, hallucinogens, and nicotine (Liu et al., 2023; Taquet et al., 2021; Wang, Wang, Wang, & Wei, 2022).

Baseline psychotropic use included antipsychotics. Suicidality indicators included suicidal ideation, suicide attempts, and intentional self-harm. Healthcare utilization included ambulatory, emergency, and inpatient visits. Depression severity was defined by diagnostic coding for single or recurrent episodes, with or without psychotic features.

A full list of covariates and their operational definitions is presented in Supplementary Table 3.

### Outcomes and follow-up

The primary outcome was a composite of all-cause hospitalization and emergency room (ER) visits, used as a proxy for clinical instability. Secondary outcomes included the individual risks of hospitalization, ER visits, suicidal behavior, manic episodes, and recurrence of MDD. Suicidal behavior was defined using ICD-10-CM diagnostic codes for suicidal ideation (R45.851), suicide attempt (X60–X84), and intentional self-harm (T14.91). All outcomes were identified based on standardized diagnosis and visit-type codes recorded during inpatient or outpatient encounters within the electronic health records of participating healthcare organizations in the TriNetX network. A complete list of codes is provided in Supplementary Table 4. Follow-up began 180 days after the index date and continued until the first occurrence of the outcome, death, or day 360, whichever came first. The follow-up period was defined as 180 days after the index event. This duration aligns with established pharmacovigilance protocols for psychostimulants. Previous large-scale observational studies, such as those evaluating cardiovascular safety and long-term developmental outcomes, have similarly utilized 6-month windows or intervals as primary observation periods (Garcia-Argibay et al., 2024; Man et al., 2023).

### Statistical analysis

Continuous variables were summarized as means with standard deviations (SDs), and categorical variables as counts and percentages. To reduce confounding, 1:1 propensity score matching (PSM) was performed using a nearest-neighbor greedy algorithm without replacement, with a caliper width of 0.1 pooled SD (Haukoos & Lewis, 2015). Matching variables included demographics, medical and psychiatric comorbidities, baseline healthcare utilization, and psychotropic medication use.

Within the TriNetX platform, once cohorts, index dates, outcomes, and covariates were defined, a covariate matrix was automatically generated for each individual based on values observed within 1 year before the index date. Propensity scores were estimated using logistic regression to model the probability of receiving methylphenidate. Patients in the methylphenidate group were then matched to patients in the control group with similar scores.

Time-to-event outcomes were analyzed using Kaplan–Meier estimators, and differences between groups were assessed using log-rank tests. Hazard ratios (HRs) with 95% confidence intervals (CIs) were estimated using Cox proportional hazards models. Proportionality assumptions were evaluated using Schoenfeld residuals. *E*-values were calculated to assess the robustness of observed associations to potential unmeasured confounding.

### Stratified analyses

Prespecified stratified analyses were conducted to examine whether the association between methylphenidate use and outcomes varied across subgroups. Analyses were stratified by age group (18–64 vs. ≥65 years), sex (male vs. female), and antidepressant class (tricyclic antidepressants [TCA], selective serotonin reuptake inhibitors [SSRI], and serotonin–norepinephrine reuptake inhibitors [SNRI]). Within each stratum, separate propensity score matching and outcome analyses were performed using the same methods as in the primary analysis.

## Results

### Patient selection

**Figure 1.**
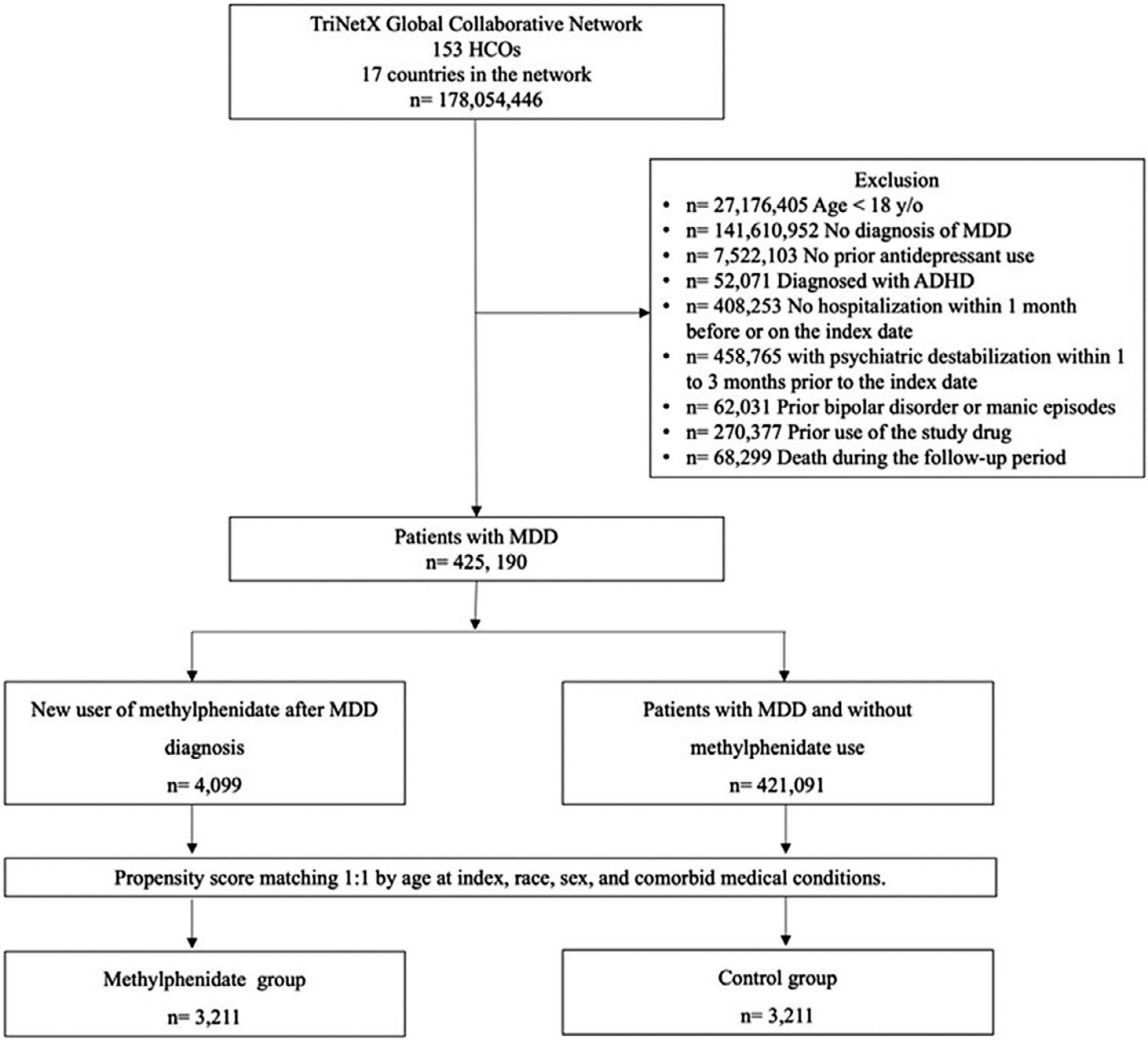
Study cohort process. ADHD, attention-deficit/hyperactivity disorder. MDD, major depressive disorder.

A total of 178,054,446 individuals were identified in the TriNetX Global Collaborative Network as of June 1, 2024. Of these, 177,629,256 were excluded for meeting at least one of the following criteria: age < 18 years; absence of a MDD diagnosis; no prior antidepressant use; diagnosis of ADHD; no hospitalization within 1 month before or on the index date; psychiatric destabilization within 1 to 3 months before the index date; history of bipolar disorder or manic episodes (ICD-10-CM F30–F31); prior use of methylphenidate; or death during the follow-up period. Among the remaining 425,190 patients with MDD, 4,099 were newly treated with methylphenidate, and 421,091 had not received methylphenidate. After 1:1 propensity score matching based on age at index, race, sex, and comorbid medical conditions, a total of 3,211 matched pairs were included in the final analysis (Figure 1).

### Demographic characteristics

Table 1 summarizes the baseline characteristics of patients in the methylphenidate and nonmethylphenidate cohorts before and after propensity score matching. Before matching, patients in the methylphenidate group were slightly older and had a higher prevalence of cardiometabolic and psychiatric comorbidities, greater use of antipsychotics, and more frequent ambulatory, emergency, and inpatient encounters. After 1:1 propensity score matching, the two cohorts were well balanced, yielding 3,211 patients in each group. Covariate balance before and after matching is illustrated in the Love plot (Supplementary Figure 4).

**Table 1.** Baseline characteristics of methylphenidate and non- methylphenidate groups before and after matching

Variables	Before matching			After matching		
	Methylphenidate group (*n* = 4,099)	Control group (*n* = 421,091)	Standardized difference	Methylphenidate group (*n* = 3,211)	Control group (*n* = 3,211)	Standardized difference
Age at index						
Mean (SD)	55.8 (20.6)	54.2 (19.9)	0.08	55.7 (20.6)	55.9 (20.2)	0.014
Sex, *n* (%)						
Female	2,379 (58.0)	265,141 (63.0)	0.101	1,858 (57.9)	1,846 (57.5)	0.008
Male	1,595 (38.9)	142,863 (33.9)	0.104	1,264 (39.4)	1,270 (39.6)	0.004
Race, *n* (%)						
White	3,073 (75.0)	312,295 (74.2)	0.019	2,416 (75.2)	2,468 (76.9)	0.038
Black or African American	360 (8.8)	38,757 (9.2)	0.015	273 (8.5)	271 (8.4)	0.002
Asian	127 (3.1)	7,268 (1.7)	0.091	102 (3.2)	97 (3.0)	0.009
Other race	141 (3.4)	12,042 (2.9)	0.033	108 (3.4)	97 (3.0)	0.019
Comorbidities, *n* (%)						
Type 2 diabetes mellitus	906 (22.1)	50,689 (12.0)	0.271	694 (21.6)	670 (20.9)	0.018
Hypertension	1,957 (47.7)	105,162 (25.0)	0.487	1,503 (46.8)	1,563 (48.7)	0.037
Hyperlipidemia	1,258 (30.7)	57,433 (13.6)	0.419	963 (30.0)	975 (30.4)	0.008
Chronic obstructive pulmonary disease	370 (9.0)	22,859 (5.4)	0.139	299 (9.3)	292 (9.1)	0.008
Chronic kidney disease	483 (11.8)	21,868 (5.2)	0.238	371 (11.6)	368 (11.5)	0.003
Ischemic heart diseases	719 (17.5)	39,922 (9.5)	0.237	572 (17.8)	544 (16.9)	0.023
Alcohol use disorder	267 (6.5)	13,729 (3.3)	0.151	172 (5.4)	164 (5.1)	0.011
Opioid use disorder	123 (3.0)	5,772 (1.4)	0.112	83 (2.6)	73 (2.3)	0.021
Cannabis use disorder	127 (3.1)	4,791 (1.1)	0.136	80 (2.5)	67 (2.1)	0.027
Cocaine ruse disorder	39 (1.0)	2,371 (0.6)	0.045	32 (1.0)	28 (0.9)	0.013
Stimulant use disorder	33 (0.8)	2,090 (0.5)	0.038	26 (0.8)	15 (0.5)	0.043
Nicotine dependence	587 (14.3)	34,967 (8.3)	0.191	459 (14.3)	426 (13.3)	0.031
Major depressive disorder, recurrent	885 (21.6)	2,143 (0.5)	0.714	149 (4.6)	134 (4.2)	0.023
Anxiety disorder	1,732 (42.3)	64,344 (15.3)	0.624	1,195 (37.2)	1,226 (38.2)	0.021
Post-traumatic stress disorder	172 (4.2)	4,045 (1.0)	0.205	99 (3.1)	94 (2.9)	0.009
Intellectual disabilities	19 (0.5)	573 (0.1)	0.061	17 (0.5)	16 (0.5)	0.004
Suicidal ideations	483 (11.8)	7,776 (1.8)	0.402	175 (5.5)	165 (5.1)	0.014
Suicidal attempt	37 (0.9)	814 (0.2)	0.096	14 (0.4)	14 (0.4)	<.001
Intentional self-harm	36 (0.9)	755 (0.2)	0.096	18 (0.6)	19 (0.6)	0.004
Medication, *n* (%)						
Antipsychotics	1,661 (40.5)	52,096 (12.4)	0.673	1,120 (34.9)	1,117 (34.8)	0.002
Aripiprazole	263 (6.4)	5,122 (1.2)	0.274	122 (3.8)	131 (4.1)	0.014
Quetiapine	425 (10.4)	11,467 (2.7)	0.313	279 (8.7)	274 (8.5)	0.006
Olanzapine	522 (12.7)	7,044 (1.7)	0.438	310 (9.7)	329 (10.2)	0.021
Visit						
Ambulatory	2,815 (68.7)	261,206 (62.0)	0.141	2,142 (66.7)	2,164 (67.4)	0.015
Emergency	1,588 (38.7)	114,543 (27.2)	0.247	1,145 (35.7)	1,117 (34.8)	0.018
Inpatient encounter	2,815 (68.7)	261,206 (62.0)	1.203	2,627 (81.8)	2,603 (81.1)	0.019

### Primary outcome

**Figure 2.**
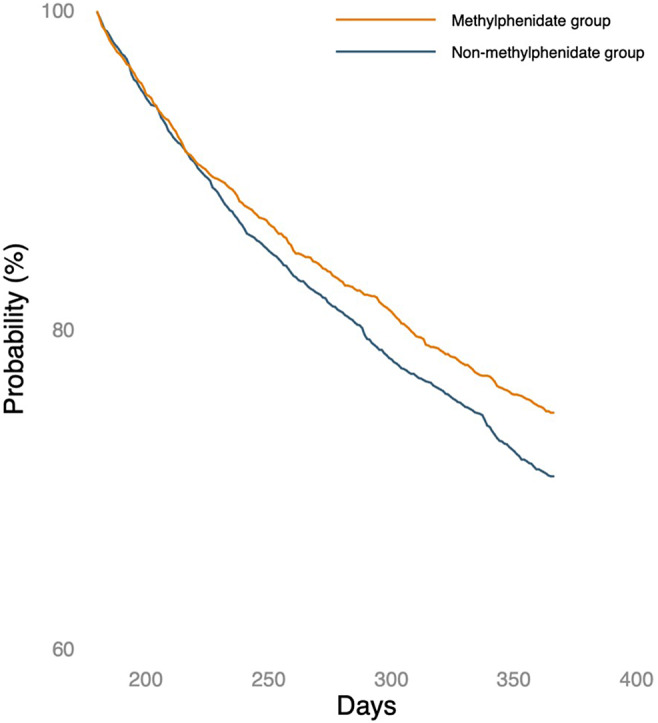
Kaplan–Meier time-to-event free curves of the composite outcome.

During follow-up, the composite outcome of hospitalization and ER visits occurred in 574 patients in the methylphenidate group and 694 patients in the control group. Methylphenidate use was associated with a significantly lower risk of the composite outcome compared with controls (HR, 0.85; 95% CI, 0.76–0.95; *p* = 0.004; *E*-value, 1.64 [95% CI lower limit, 1.29], Table 2), a finding consistent with the Kaplan–Meier analysis (Figure 2).

**Table 2.** Hazard ratio of outcomes between methylphenidate and non-methylphenidate groups

Outcome	Methylphenidate group(*n* = 3,211)	Control group(*n* = 3,211)	HR (95% CI)	*p*-value	*E*-value (95% LCL)
	Events (*n*)	Events (*n*)			
Primary outcome					
Composite outcome of hospitalization and emergency room visits	574	694	0.85 (0.76, 0.95)	0.004	1.64 (1.29)
Secondary outcomes					
Hospitalization	385	435	0.92 (0.81, 1.06)	0.251	1.38 (1.31)
Emergency room visits	343	397	0.89 (0.78, 1.04)	0.137	1.48 (1.23)
Suicidal behavior	27	24	1.17 (0.68, 2.04)	0.558	1.64 (2.30)
Manic episodes	25	28	0.52 (0.05, 5.76)	0.589	3.24 (10.9)
MDD recurrence	84	71	1.25 (0.91, 1.71)	0.171	1.80 (1.43)

MDD, major depressive disorder.

### Secondary outcomes

For the individual outcomes, hospitalization occurred in 385 patients in the methylphenidate group and 435 patients in the control group, showing no significant difference (HR, 0.92; 95% CI, 0.81–1.06; *p* = 0.25). ER visits occurred in 343 patients in the methylphenidate group and 397 in the control group, also without a significant association (HR, 0.89; 95% CI, 0.78–1.04; *p* = 0.14) (Table 2).

Regarding psychiatric outcomes showed no significant differences between groups. Suicidal behavior occurred in 27 patients in the methylphenidate group and 24 patients in the control group (HR, 1.17; 95% CI, 0.68–2.04; *p* = 0.56). Manic episodes occurred in 25 and 28 patients, respectively, without statistical significance (HR, 0.52; 95% CI, 0.05–5.76; *p* = 0.59). MDD recurrence occurred in 84 patients in the methylphenidate group and 71 patients in the control group, also not statistically significant (HR, 1.25; 95% CI, 0.91–1.71; *p* = 0.17) (Table 2).

### Subgroup analyses

**Figure 3.**
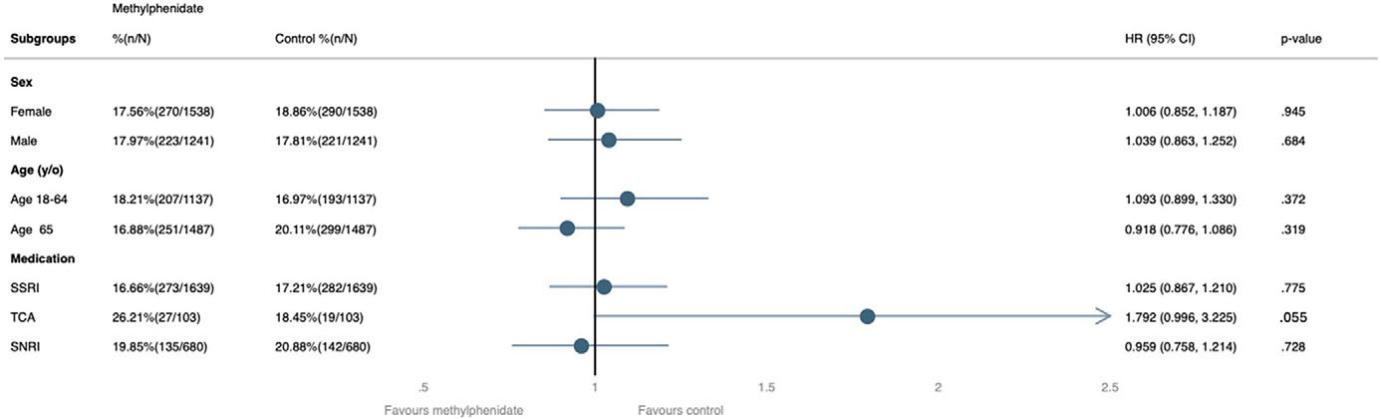
Subgroup analyses of the composite outcome risk. CI, confidence interval; HR, hazard ratio; SNRI, serotonin-norepinephrine reuptake inhibitor; SSRI, selective serotonin reuptake inhibitor; TCA, tricyclic antidepressant; y/o, years old.

Subgroup analyses of the composite primary outcome (hospitalization and ER visits) showed no significant differences across sex, age, and concomitant antidepressant use (Figure 3). Among sex subgroups, there were no significant differences between groups (female: HR, 1.01; 95% CI, 0.85–1.19; *p* = 0.95; male: HR, 1.04; 95% CI, 0.86–1.25; *p* = 0.68). Across age groups, the results were not significant (18–64 years: HR, 1.09; 95% CI, 0.90–1.33; *p* = 0.37; ≥65 years: HR, 0.92; 95% CI, 0.78–1.09; *p* = 0.32). When stratified by concomitant antidepressant use, none of the subgroups showed a significant association (SSRIs: HR, 1.03; 95% CI, 0.87–1.21; *p* = 0.78; TCAs: HR, 1.79; 95% CI, 0.99–3.23; *p* = 0.06; SNRIs: HR, 0.96; 95% CI, 0.76–1.21; *p* = 0.73).

For hospitalization (Supplementary Figure 1), no significant differences were observed across sex (female: HR, 1.20; 95% CI, 0.97–1.48; *p* = 0.10; male: HR, 1.06; 95% CI, 0.84–1.33; *p* = 0.62), age (18–64 years: HR, 1.27; 95% CI, 0.98–1.64; *p* = 0.07; ≥65 years: HR, 1.09; 95% CI, 0.88–1.34; *p* = 0.44), or antidepressant use (SSRIs: HR, 1.19; 95% CI, 0.96–1.48; *p* = 0.12; TCAs: HR, 1.21; 95% CI, 0.60–2.45; *p* = 0.59; SNRIs: HR, 1.12; 95% CI, 0.83–1.51; *p* = 0.45).

For ER visits (Supplementary Figure 2), no significant associations were detected across sex (female: HR, 1.06; 95% CI, 0.86–1.30; *p* = 0.61; male: HR, 1.20; 95% CI, 0.94–1.55; *p* = 0.15), age (18–64 years: HR, 1.02; 95% CI, 0.80–1.30; *p* = 0.86; ≥65 years: HR, 0.94; 95% CI, 0.76–1.16; *p* = 0.55), and antidepressant use (SSRIs: HR, 1.03; 95% CI, 0.84–1.27; *p* = 0.77; TCAs: HR, 1.52; 95% CI, 0.74–3.13; *p* = 0.25; SNRIs: HR, 0.99; 95% CI, 0.73–1.33; *p* = 0.92).

For suicidal behavior (Supplementary Figure 3), no subgroup showed a statistically significant association, including sex (female: HR, 1.62; 95% CI, 0.73–3.61; *p* = 0.23; male: HR, 1.47; 95% CI, 0.56–3.85; *p* = 0.44), age (18–64 years: HR, 0.85; 95% CI, 0.45–1.63; *p* = 0.63; ≥65 years: HR, 1.37; 95% CI, 0.37–5.10; *p* = 0.64), and antidepressant use (SSRIs: HR, 1.21; 95% CI, 0.58–2.55; *p* = 0.61; SNRIs: HR, 1.00; 95% CI, 0.37–2.65; *p* = 0.99), with insufficient data for TCAs.

## Discussion

To the best of our knowledge, this is the first large-scale, multi-institutional study to evaluate the real-world safety of methylphenidate in patients with MDD. We found that methylphenidate use was associated with a significantly lower risk of the composite endpoint of all-cause hospitalization and ER visits. No significant differences were observed in secondary outcomes, including hospitalization, emergency visits, suicidal behavior, manic episodes, or depression recurrence. Subgroup analyses stratified by sex, age, and concomitant antidepressant class likewise revealed no differential risks, reinforcing the robustness of these results.

Our main finding was that methylphenidate use was associated with a significantly lower risk of the composite outcome of hospitalization and ER visits, providing reassurance that stimulant treatment does not destabilize psychiatric illness in patients with MDD. Although early reports raised concerns regarding stimulant-induced mania or suicidality (Arun & Sahni, 2014; Ross, 2006), subsequent clinical studies have demonstrated that adjunctive stimulant therapy can alleviate fatigue, apathy, and cognitive slowing in depression without increasing adverse psychiatric outcomes (Hardy, 2009; Lavretsky et al., 2015b; Pary et al., 2015). By blocking dopamine and norepinephrine reuptake, methylphenidate counteracts psychomotor slowing and residual anergia, thereby improving motivation, concentration, and adherence to outpatient care (Pary et al., 2015). This enhancement of energy and cognitive function may help patients better cope with stressors and avoid decompensation severe enough to require emergency or inpatient treatment (Kerr et al., 2012; Smith, Kahlon, Brown, & Britt, 2021). Supporting this, a randomized controlled trial by Lavretsky et al. (2015a)) further showed that combined treatment with citalopram and methylphenidate produced greater improvement in mood, well-being, and remission rates compared with citalopram alone in patients with geriatric depression (Lavretsky et al., 2015b). In addition, large registry studies and meta-analyses in ADHD populations have suggested that stimulant treatment is associated with lower rates of psychiatric hospitalization and suicidal behavior (W. j. Liu et al., 2020; Taipale et al., 2024; Zhang et al., 2025). Collectively, stimulant augmentation may address therapeutic gaps left by conventional antidepressants and contribute to greater overall stability in patients with MDD.

Importantly, we found no evidence that methylphenidate increased suicidal behavior in patients with MDD. This finding directly addresses long-standing clinical concerns that stimulants might exacerbate impulsivity or provide the energy to act on suicidal thoughts (Anestis et al., 2014; Kerkeni & Pons, 2024). Prior pharmacoepidemiologic studies in ADHD populations have consistently shown that stimulant exposure is not associated with higher rates of suicide attempts; in fact, some large registry analyses reported a modest reduction in suicidal behavior among individuals adherent to stimulant therapy (Anestis et al., 2014; Chang et al., 2020). A systematic review and network meta-analysis of randomized controlled trials of stimulant-type medications for depression further indicated that methylphenidate was generally well tolerated, with no signal of increased suicidality or impulsivity, although the evidence base was limited by small sample sizes and incomplete reporting (Bahji & Mesbah-Oskui, 2021). Consistent with these findings, a large Danish nationwide self-controlled register study of patients with depression reported that initiation of methylphenidate was associated with a 54% reduction in self-harm or suicide attempts, without an increase in psychiatric admissions or inpatient days (Rohde, Brink, Østergaard, & Nielsen, 2020). Our study extends this literature by focusing on a more specific population of patients with unipolar depression who required hospitalization, while excluding those with comorbid ADHD, thereby reducing diagnostic heterogeneity. In this clearly defined cohort, suicidal behavior was rare and occurred at similar rates between methylphenidate users and matched controls. Clinically, this provides reassurance that methylphenidate can be considered as an adjunctive treatment option without increasing suicidality risk, provided that patients continue to receive standard monitoring for suicidal ideation as part of routine care.

In evaluating the risk of manic episodes, our study found no evidence that methylphenidate precipitated mania in patients with MDD. This observation is consistent with large registry studies showing that methylphenidate initiation is not associated with increased manic switching in bipolar populations, particularly when used with appropriate mood-stabilizing strategies (Jefsen, Østergaard, & Rohde, 2023; Pary et al., 2015; Viktorin et al., 2017). Moreover, a review by Pary et al. (2015) on unipolar depression did not identify manic conversion as a significant adverse event. Taken together, these findings suggest that in carefully screened patients with unipolar depression and no history of mania, methylphenidate does not appear to increase the risk of manic episodes and may be considered a safe adjunctive option.

Regarding the recurrence of MDD, our study found no evidence that methylphenidate increased the risk of relapse during the 1-year follow-up period. This finding is clinically relevant because a central concern with stimulant augmentation is whether short-term symptomatic benefits might be offset by long-term mood instability. In the acute phase, stimulants may alleviate residual symptoms such as psychomotor slowing, low motivation, and cognitive inefficiency that are not fully addressed by conventional antidepressants, thereby potentially delaying rather than accelerating relapse (Blier, 2013). Nevertheless, the long-term trajectory beyond 1 year remains uncertain. A meta-analysis by Oliva et al. has evaluated the safety of stimulants in adult populations and incorporated some trials of methylphenidate in treatment-resistant depression, but these studies were generally short in duration (6–12 weeks) and did not address recurrence outcomes (Oliva et al., 2025). In contrast, our study provides a 1-year follow-up, a relative strength compared with prior short-term trials. Nonetheless, data on longer-term safety remain limited, and prospective studies are needed to clarify outcomes beyond 1 year.

There are limitations to our study. First, as with all observational research, causal inference is limited by the possibility of residual confounding. Although we utilized propensity score matching to balance observable characteristics and employed strict cohort definitions to harmonize baseline severity, unmeasured factors, such as lifestyle behaviors or socioeconomic status not captured in EHRs may still influence the results. Therefore, our findings should be interpreted as associations rather than definitive causal relationships. Second, some outcomes, such as manic episodes and suicidal behavior, were rare, which limited statistical power. Nevertheless, our large sample size and consistent results across related endpoints reduce the likelihood that a clinically meaningful effect was overlooked. Third, the follow-up period was limited to 1 year, which is longer than most prior stimulant augmentation trials, but insufficient to clarify the long-term trajectory of methylphenidate use in MDD. Fourth, regarding the duration of exposure, the TriNetX dataset relies on prescription orders and does not provide granular information on medication adherence or the exact duration of continuous use. Consequently, we could not stratify risks based on cumulative dose or treatment length. However, we employed an intention-to-treat framework based on the index prescription. This design captures the risks associated with the clinical decision to initiate methylphenidate, minimizing the selection bias inherent in analyses restricted to long-term adherers, and ensures our findings reflect real-world clinical scenarios. Another limitation concerns the generalizability of our findings regarding both the study population and the spectrum of adverse outcomes. The cohort was restricted to patients with an inpatient diagnosis of depression, likely representing a more severe phenotype; therefore, the findings may not apply to outpatients with mild-to-moderate depression managed exclusively in ambulatory settings. Similarly, our outcome assessment focused on serious adverse events severe enough to require hospitalization. Finally, this study did not specifically evaluate the potential for medication abuse or misuse. Given the pharmacological profile of methylphenidate, abuse potential remains an important safety concern that could not be fully captured within the current study. Despite these limitations, the large sample size, rigorous cohort definition, and extended observation period strengthen confidence in the robustness of our findings.

## Conclusion

Methylphenidate use in patients with MDD was associated with a significantly lower risk of the composite outcome of hospitalization and ER visits and was not linked to increased risks of hospitalization, emergency visits, suicidality, mania, or relapse. These findings suggest that methylphenidate augmentation may be a safe option for managing residual symptoms, although prospective studies are needed to establish its long-term safety and effectiveness.

## Supporting information

10.1017/S0033291726103845.sm001 Liu et al. supplementary material Liu et al. supplementary material

## Supplementary material

The supplementary material for this article can be found at http://doi.org/10.1017/S0033291726103845.
