# Supplementary material for: Psychiatric safety of methylphenidate in adults with major depressive disorder: a 1-year retrospective cohort study of 6,422 patients
Source: Psychol Med. 2026 May 5;56:e130. doi: 10.1017/S0033291726103845 (PMC13161795; doi:10.1017/S0033291726103845)

**Supplementary Material**

**Psychiatric Safety of Methylphenidate in Adults With Major Depressive Disorder: A Real-World Cohort Analysis of 6,422 Patients**

Ting-Hui Liu ^1^, Ya-Lin Huang ^1^, Jheng-Yan Wu ^2^, Chien-Ho Lin ^1^, Fong-Lin Jang^1§^, Chih-Cheng Lai ^4,5§^

1. Department of Psychiatry, Chi Mei Medical Center, Tainan, Taiwan
2. Department of Nutrition, Chi Mei Medical Center, Tainan, Taiwan
3. Department of Public Health, College of Medicine, National Cheng Kung University, Tainan, Taiwan
4. Division of Hospital Medicine, Department of Internal Medicine, Chi Mei Medical Center, Tainan, Taiwan
5. School of Medicine, College of Medicine, National Sun Yat-sen University, Kaohsiung, Taiwan

Supplementary Table 1. Full diagnostic code definitions for cohort selection

| **Data type** | **Code system:Code** | **Term / Description** |
| --- | --- | --- |
| Demographics | Age | ≥ 18 years |
| Medication | NLM:RXNORM:6901 | Methylphenidate |
| Diagnosis | ICD10CM:F32.0, F32.1, F32.2, F32.3, F32.8, F32.9, F32.A; ICD9CM:296.2; ICD10CM:F33.0–F33.3, F33.8, F33.9; ICD9CM:296.3 | Major depressive disorder (single/recurrent episodes, specified/unspecified) |
| Medication | NLM:ATC:N06A | Antidepressants |
| Visit / Diagnosis | HL7V3.0:IMP; ICD10CM:X71–X83, R45.851, T14.91 | Inpatient encounter; suicidal ideations; intentional self-harm; suicide attempt |
| Diagnosis / | ICD10CM:F30, F31; | Manic episode; bipolar disorder |
| Procedure / Medication | CPT:80178; RxNorm:40254, 2002 | lithium; valproate; carbamazepine |
| Demographics | Deceased | Deceased |
| Visit | HL7V3.0:IMP | Inpatient encounter |
| Diagnosis | UMLS:ICD10CM:F90 | Attention-deficit hyperactivity disorders |

Supplementary Table 2. Full code for index event definitions

| **Cohort** | **Requirement** | **Data type** | **Code system:Code** | **Description** |
| --- | --- | --- | --- | --- |
| **Methylphenidate group** | Must have | Medication | NLM:RXNORM:6901 | Methylphenidate |
|  | Must have | Diagnosis | ICD10CM: F32.0, F32.1, F32.2, F32.3, F32.8, F32.9, F32.A; ICD9CM:296.2; ICD10CM: F33.0, F33.1, F33.2, F33.3, F33.8, F33.9; ICD9CM:296.3 | Major depressive disorder (single or recurrent episodes, mild to severe, with or without psychotic features, or unspecified; depression) |
|  | A major depressive disorder diagnosis was required within one month prior to the first methylphenidate prescription. | | | |
| **Control Group** | Must have | Diagnosis | ICD10CM: F32.0, F32.1, F32.2, F32.3, F32.8, F32.9, F32.A; ICD9CM:296.2; ICD10CM: F33.0, F33.1, F33.2, F33.3, F33.8, F33.9; ICD9CM:296.3 | Major depressive disorder (single or recurrent episodes, mild to severe, with or without psychotic features, or unspecified; depression |

Supplementary Table 3. Covariates and corresponding code definitions

| **Category** | **Code (System)** | **Code Description** |
| --- | --- | --- |
| **Demographics** | AI | Age at Index |
|  | F | Female |
|  | M | Male |
|  | 2106-3 | White |
|  | 2054-5 | Black or African American |
|  | 2028-9 | Asian |
|  | 2131-1 | Other Race |
|  | UNK | Unknown Race |
| **Comorbidities** | E11 (ICD-10-CM) | Type 2 diabetes mellitus |
|  | I10 (ICD-10-CM) | Essential (primary) hypertension |
|  | E78.5 (ICD-10-CM) | Hyperlipidemia, unspecified |
|  | J44 (ICD-10-CM) | Other chronic obstructive pulmonary disease |
|  | N18 (ICD-10-CM) | Chronic kidney disease (CKD) |
|  | I20–I25 (ICD-10-CM) | Ischemic heart diseases |
|  | F10 (ICD-10-CM) | Alcohol-related disorders |
|  | F11 (ICD-10-CM) | Opioid-related disorders |
|  | F12 (ICD-10-CM) | Cannabis-related disorders |
|  | F14 (ICD-10-CM) | Cocaine-related disorders |
|  | F15 (ICD-10-CM) | Other stimulant-related disorders |
|  | F17 (ICD-10-CM) | Nicotine dependence |
|  | F33 (ICD-10-CM) | Major depressive disorder, recurrent |
|  | F40–F48 (ICD-10-CM) | Anxiety, dissociative, stress-related, somatoform, and other nonpsychotic mental disorders |
|  | F43.1 (ICD-10-CM) | Post-traumatic stress disorder |
|  | F70–F79 (ICD-10-CM) | Intellectual disabilities |
|  | R45.851 (ICD-10-CM) | Suicidal ideations |
|  | T14.91 (ICD-10-CM) | Suicide attempt |
|  | X71–X83 (ICD-10-CM) | Intentional self-harm |
| **Medication** | N05A (ATC) | Antipsychotics |
|  | 89013 (RxNorm) | Aripiprazole |
|  | 51272 (RxNorm) | Quetiapine |
|  | 61381 (RxNorm) | Olanzapine |
|  | 1658314 (RxNorm) | Brexpiprazole |
| **Visit** | HL7V3.0:IMP | Visit: Inpatient encounter |
|  | \| HL7V3.0:AMB \| \| --- \| | \| Visit: Ambulatory encounter \| \| --- \| |
|  | HL7V3.0:EMER | Visit: Emergency encounter |

Supplementary table 4. Diagnostic, Visit, and Procedural Codes Used in the Definition of Outcomes

| **Category** | **Code (System)** | **Description** |
| --- | --- | --- |
| **Suicidal behavior** | ICD10CM:R45.851 | Suicidal ideations |
|  | ICD10CM:T14.91 | Suicide attempt |
|  | ICD10CM:X71–X83 | Intentional self-harm |
| **Hospitalization** | HL7V3.0:VisitType:IMP | Visit: Inpatient encounter |
| **Mania** | ICD10CM:F30 | Manic episode |
| **Major depressive disorder, recurrence** | ICD10CM:F33.0 | Major depressive disorder, recurrent, mild |
|  | ICD10CM:F33.1 | Major depressive disorder, recurrent, moderate |
|  | ICD10CM:F33.2 | Major depressive disorder, recurrent, severe without psychotic features |
|  | ICD10CM:F33.3 | Major depressive disorder, recurrent, severe with psychotic symptoms |
|  | ICD10CM:F33.9 | Major depressive disorder, recurrent, unspecified |
|  | ICD9CM:296.3 | Major depressive disorder, recurrent episode |
| **Emergency room visit** | HL7V3.0:VisitType:EMER | Visit: Emergency encounter |
| **Inpatient + Emergency room visit** | HL7V3.0:VisitType:EMER | Visit: Emergency encounter |
|  | HL7V3.0:VisitType:IMP | Visit: Inpatient encounter |

Supplementary figure 1. Subgroup analyses of the risk of emergency room visits comparing patients treated with methylphenidate and matched controls


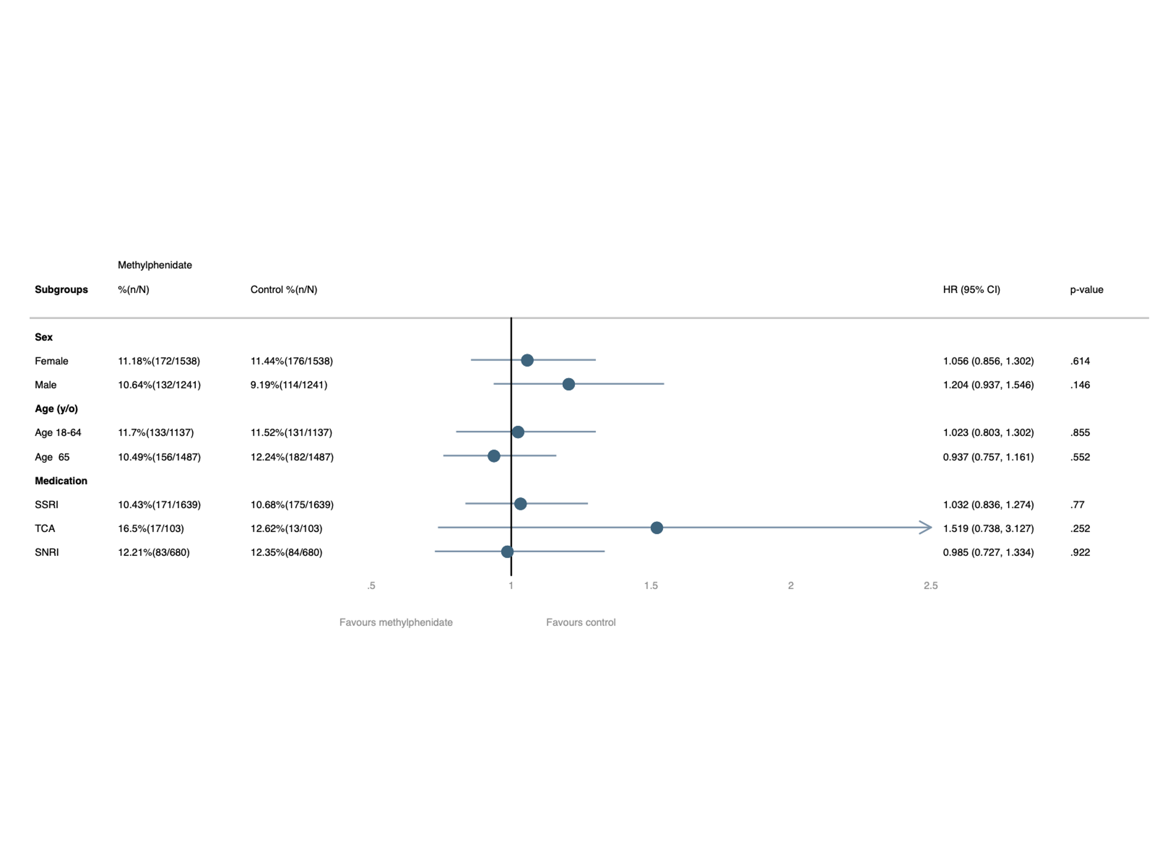


Supplementary figure 2. Subgroup analyses of the risk of hospitalization comparing patients treated with methylphenidate and matched controls


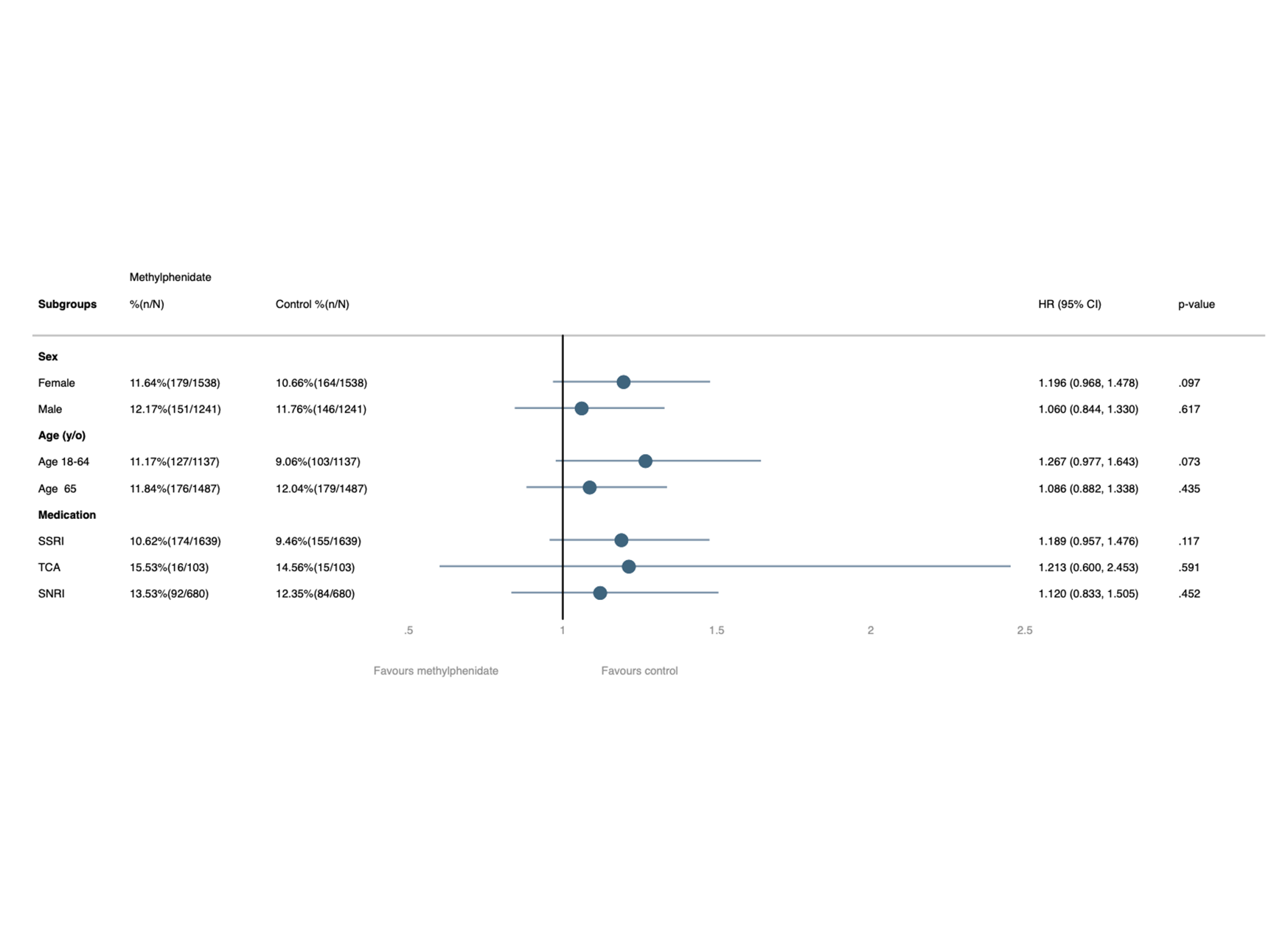


Supplementary figure 3. Subgroup analyses of the risk of suicidal behavior comparing patients treated with methylphenidate and matched controls


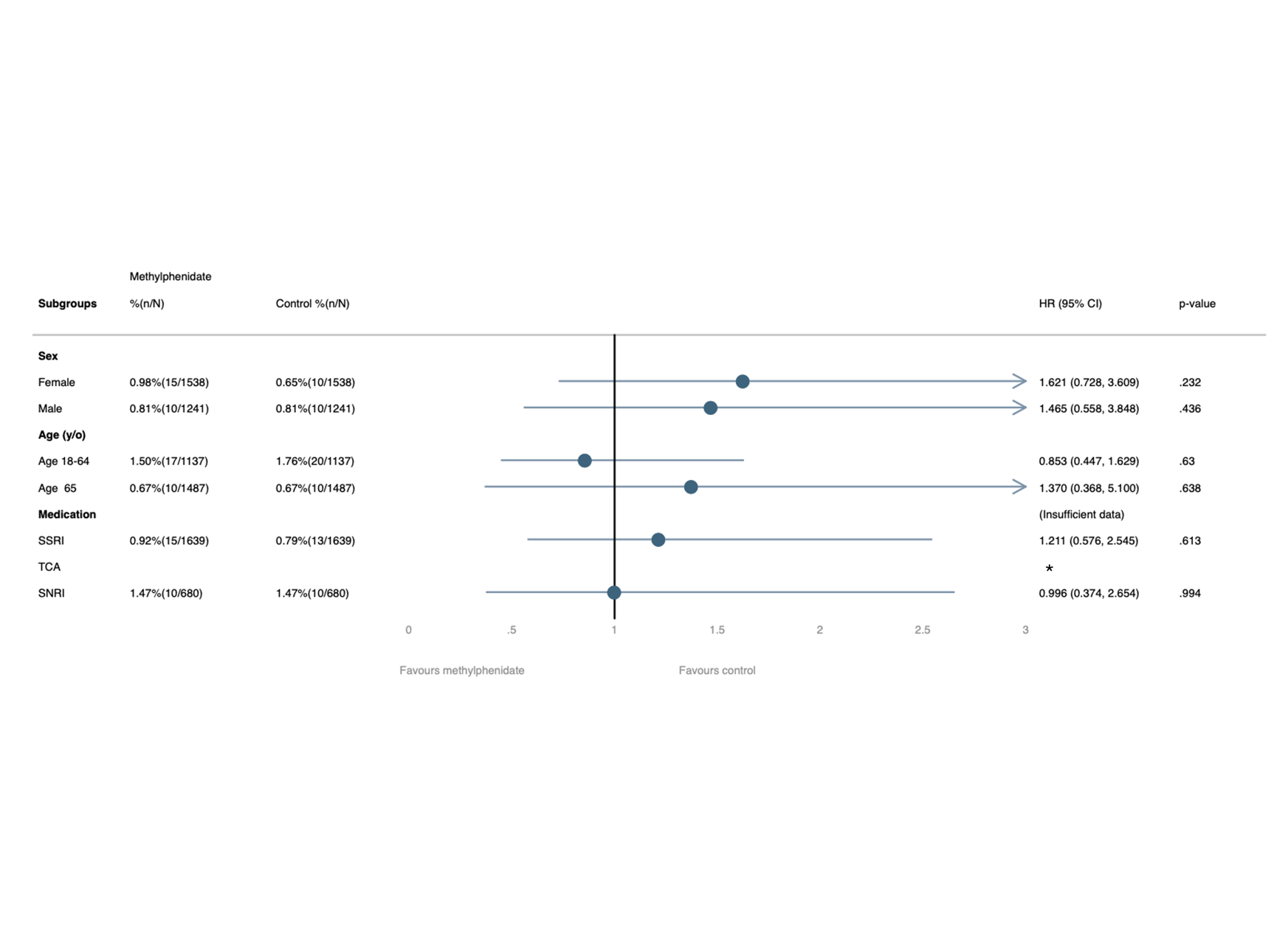


Supplementary figure 4. Love plot for Propensity Score Matching


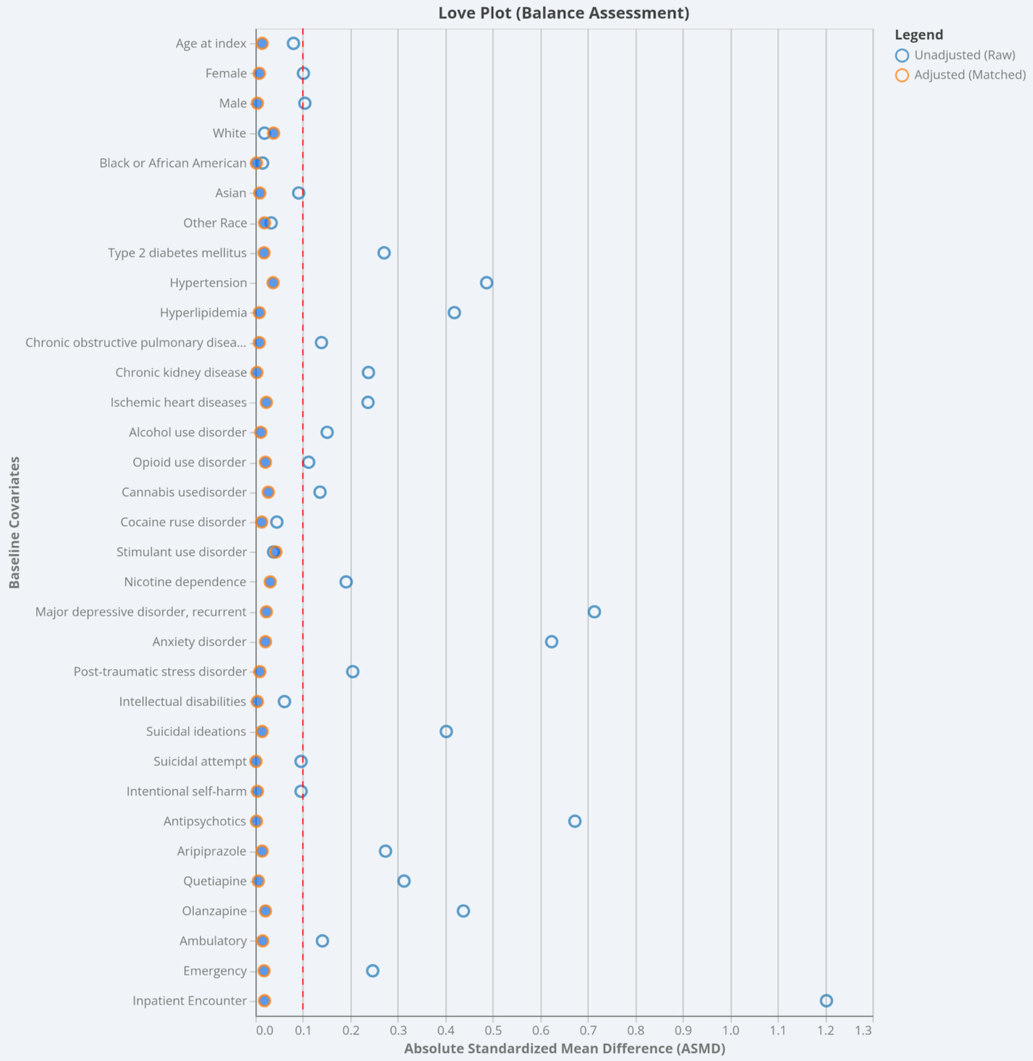

Supplement: Liu et al. supplementary material [file S0033291726103845sup001.docx]
